# Aurora Kinase A-YBX1 Synergy Fuels Aggressive Oncogenic Phenotypes and Chemoresistance in Castration-Resistant Prostate Cancer

**DOI:** 10.3390/cancers12030660

**Published:** 2020-03-12

**Authors:** Kumar Nikhil, Asif Raza, Hanan S. Haymour, Benjamin V. Flueckiger, Jiachong Chu, Kavita Shah

**Affiliations:** Department of Chemistry and Purdue University Center for Cancer Research, Purdue University, 560 Oval Drive, West Lafayette, IN 47907, USA; knikhil@purdue.edu (K.N.); razaasif.biotech@gmail.com (A.R.); hhaymour@purdue.edu (H.S.H.); bfluecki@purdue.edu (B.V.F.); chu95@purdue.edu (J.C.)

**Keywords:** Aurora kinase A (AURKA), YBX1, epithelial to mesenchymal transition, therapy, prostate cancer, CRPC

## Abstract

Multifunctional protein YBX1 upregulation promotes castration-resistant prostate cancer (CRPC). However, YBX1 protein abundance, but not its DNA status or mRNA levels, predicts CRPC recurrence, although the mechanism remains unknown. Similarly, the mechanism by which YBX1 regulates androgen receptor (AR) signaling remains unclear. We uncovered the first molecular mechanism of YBX1 upregulation at a post-translational level. YBX1 was identified as an Aurora Kinase-A (AURKA) substrate using a chemical screen. AURKA phosphorylates YBX1 at two key residues, which stabilizes it and promotes its nuclear translocation. YBX1 reciprocates and stabilizes AURKA, thereby initiating a synergistic loop. Notably, phospho-resistant YBX1 is dominant-negative and fully inhibits epithelial to mesenchymal transition, chemoresistance, drug-resistance and tumorigenesis in vivo. Unexpectedly, we further observed that YBX1 upregulates AR post-translationally by preventing its ubiquitylation, but not by increasing its transcription as reported before. Uncovering YBX1-mediated AR stabilization is highly significant due to AR’s critical role in both androgen-sensitive prostate cancer and CRPC. As YBX1 inhibitors are unknown, AURKA inhibitors provide a potent tool to degrade both YBX1 and AR simultaneously. Finally, this is the first study to show a reciprocal loop between YBX1 and its kinase, indicating that their concomitant inhibition will be act synergistically for CRPC therapy.

## 1. Introduction

Prostate cancer (PCa) is one of the most common cancer among American men [1]. Initially prostate tumors can be treated effectively by radiation, medical or surgical castration. However, treatment fails within a couple of years in virtually all patients, giving rise to castration-resistant prostate cancer (CRPC) [2]. Second generation androgen deprivation therapy (ADT) and chemotherapy agents have increased overall survival, but they remain palliative options. Therefore, it is imperative to uncover underlying molecular mechanisms and identify effective molecular targets to prevent the progression to CRPC and/or eradicate these tumors efficiently.

Aurora Kinase A (AURKA) is overexpressed in almost all PCa tumors, including CRPC [3,4,5]. AURKA inhibition synergizes with radiation therapy in CRPC cells, underscoring its fatal role in CRPC [6]. Nevertheless, AURKA inhibition has shown several adverse effects in clinical trials, presumably due to its inhibition in normal tissues. Thus, instead of targeting AURKA, selective targeting of its oncogenic substrates is a viable possibility for creating effective drugs with negligible side effects. 

This study focuses on one such AURKA substrate- Y-box binding protein-1 (YBX1 aka YB1), which we discovered using our chemical genetic methodology [7,8,9,10]. This technique utilizes a kinase of interest with an engineered pocket in the active site, which preferentially uses an orthogonal ATP analog with large groups at N-6-position (e.g., N^6^-(phenethyl) ATP) [11]. These orthogonal ATP analogs are marginally accepted by endogenous kinases, allowing unprejudiced identification of the direct targets of the kinase of interest on a proteome-wide screen [12,13,14,15,16,17,18]. We previously reported an AURKA mutant (named as AURKA-as7) that competently utilizes N-6-Phenethyl-ATP (PE-ATP), and transfers the ^32^P-γ-phosphate tag to its substrates. Using this approach, we identified several novel AURKA substrates such as ALDH1A1, PHLDA1, TWIST1 and LIMK2 [7,8,9,10]. 

YBX1 is a multifunctional protein, which functions as a transcription factor in the nucleus, and as a RNA-binding factor in the cytoplasm [19]. YBX1 is highly expressed in many cancers, including PCa. Importantly, YBX1 is upregulated after ADT [20] and promotes CRPC via androgen receptor (AR) and ARv7 overexpression, epithelial to mesenchymal transition (EMT) and chemoresistance [21,22,23,24]. EMT and cancer stem cell (CSCs) phenotypes are critical for CRPC progression [25]. Castration leads to EMT and CSC phenotypes, causing metastasis [26]. As AURKA overexpression triggers enhanced chemoresistance and EMT [9,10], we hypothesized that AURKA stimulates EMT, CSC phenotypes and chemoresistance by upregulating YBX1.

## 2. Results

### 2.1. AURKA Directly Phosphorylates YBX1 at T62 and S102

**Figure 1:**
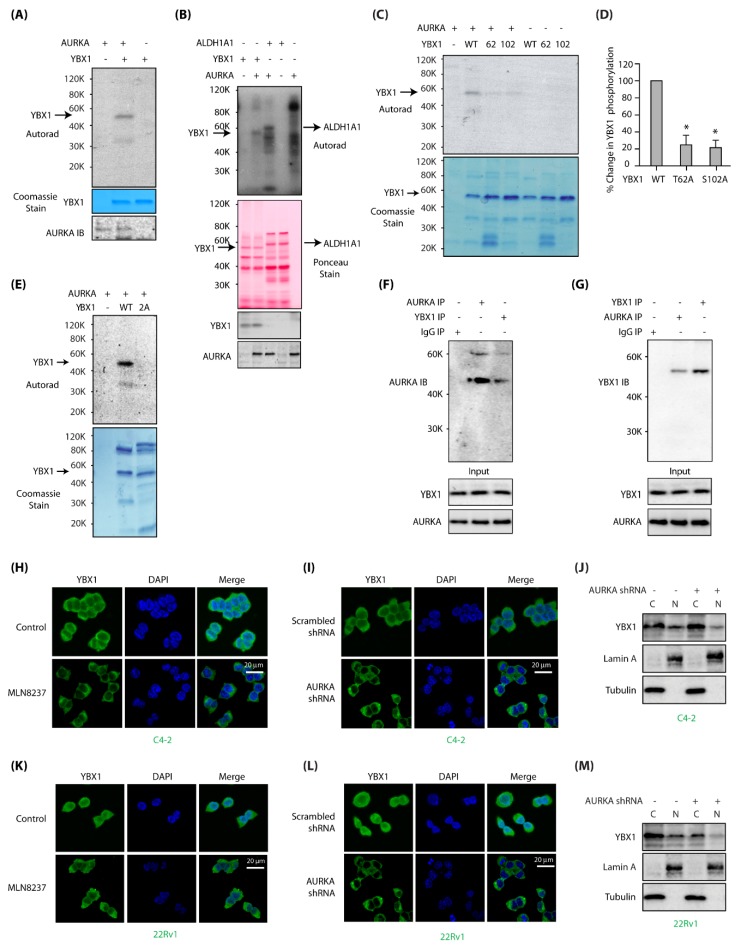
Aurora Kinase-A (AURKA) associates and directly phosphorylates Y-box binding protein-1 (YBX1) at two sites and regulates its localization in C4-2 and 22Rv1 cells. (**A**) YBX1 is a substrate of AURKA in vitro. AURKA-TPX2 was mixed with either [^32^P]ATP (lane 1), or 6x-His–YBX1 and [^32^P]ATP (lane 2) in a kinase assay for 30 min. Lane 3 shows YBX1 mixed with [^32^P]ATP. (**B**) YBX1 shows comparable phosphorylation levels as ALDH1A1, a known AURKA substrate. YBX1 and ALDH1A1 were subjected to kinase assay with AURKA and [^32^P]ATP (lanes 2 and 3, respectively). Lanes 1 and 4 are controls with YBX1 and ALDH1A1, respectively, with [^32^P]ATP. Lane 5 shows AURKA with [^32^P]ATP. (**C**) AURKA phosphorylates YBX1 at T62 and S102. Phospho-resistant YBX1 single mutants were exposed to an in vitro kinase assay using AURKA-TPX2 and [^32^P] ATP (lanes 3 and 4, respectively). WT YBX1 with [^32^P] ATP was used as a positive control (lane 2). Lanes 5, 6 and 7 show WT, T62 and S102 mutants exposed to [^32^P] ATP, respectively in the absence of AURKA. (**D**) Histogram shows % change in phosphorylation of WT and phospho-dead single mutants of YBX1 from three independent experiments. (**E**) T62 and S102 are the only AURKA sites on YBX1, as 2A-YBX1 mutant is not phosphorylated by AURKA (lane 3). Lane 2 shows WT YBX1 with AURKA and [^32^P] ATP. (**F**) YBX1 associates with AURKA in cells. YBX1 IP was conducted in C4-2 cells, and AURKA levels analyzed (lane 3). IgG (lane 1) and AURKA (lane 2) were used as negative and positive controls, respectively. (**G**) AURKA binds YBX1 in C4-2 cells. AURKA IP was conducted in C4-2 cells, and YBX1 levels analyzed (lane 2). IgG (lane 1) and YBX1 (lane 3) were used as negative and positive controls, respectively. (**H**) AURKA inhibition using MLN8237 inhibits nuclear translocation of YBX1 in C4-2 cells. C4-2 cells were treated with 1 μM MLN8237 for 12 h, and AURKA subcellular localization analyzed using AURKA-specific antibody (green). DAPI is shown in blue. (**I**) AURKA depletion inhibits nuclear translocation of YBX1. C4-2 cells were exposed to control or AURKA shRNA for 30h, fixed and stained with YBX1 antibody (green) or DAPI (blue). >100 cells were analyzed from multiple random frames. (**J**) Subcellular fractionation of scrambled shRNA-treated and AURKA shRNA-treated C4-2 cells confirms reduced nuclear translocation of YBX1 upon AURKA depletion. C4-2 cells were exposed to control or AURKA shRNA for 30 h prior to fractionation. (**K**) AURKA inhibition using MLN8237 prevents nuclear translocation of YBX1 in 22Rv1 cells. 22Rv1 cells were treated with 1 μM MLN8237 for 12 h, and AURKA subcellular localization analyzed using AURKA-specific antibody (green). (**L**) AURKA depletion inhibits nuclear translocation of YBX1 in 22Rv1 cells. 22Rv1 cells were exposed to control or AURKA shRNA for 30 h, fixed and stained with YBX1 antibody (green) or DAPI (blue). (**M**) Subcellular fractionation confirms reduced nuclear translocation of YBX1 upon AURKA depletion in 22Rv1 cells. 22Rv1 cells were exposed to control or AURKA shRNA for 30 h prior to fractionation.

To confirm the data obtained from our screen, we performed an in vitro kinase assay to assess whether YBX1 was indeed phosphorylated by AURKA. As shown in Figure 1A, YBX1 is a direct substrate of AURKA. We further compared AURKA-mediated YBX1 phosphorylation to another known AURKA substrate ALDH1A1 [10], both of which showed comparable phosphorylation levels (Figure 1B). To identify the phosphorylation sites on YBX1, we focused on phosphorylation consensus sequence of AURKA [27], which suggested T62 and S102 as potential sites on YBX1. The corresponding phosphorylation-dead mutants, T62A and S102A, were generated and used in an in vitro kinase assay with AURKA/TPX2. AURKA phosphorylates both T62 and S102 sites on YBX1 (Figure 1C). Figure 1D shows relative phosphorylation levels of WT, T62A and S102A-YBX1 mutants from three independent experiments. We also performed a kinase assay using the corresponding phosphorylation-dead double mutant (T62A, S102A, signified as 2A), which showed no phosphorylation, confirming that T62 and S102 are the only sites phosphorylated by AURKA (Figure 1E). 

### 2.2. AURKA Associates with YBX1 and Promotes Its Nuclear Localization

Kinases often associate with their substrates, which prompted us to investigate potential binding of AURKA with YBX1 in C4-2 cells. YBX1 immune complex indeed pulled down AURKA (Figure 1F, lane 3) and AURKA immune complex brought down YBX1 (Figure 1G), confirming that these proteins associate with each other in PCa cells.

Nuclear localization of YBX1 correlates with poor prognosis in many cancers including PCa [28]. In C4-2 cells, YBX1 was both cytoplasmic and nuclear (Figure 1H,I). However, when AURKA was inhibited using AURKA-specific inhibitor MLN8237 (Alisertib) or knocked-down using AURKA shRNA, YBX1 was entirely cytoplasmic, indicating that AURKA promotes nuclear translocation of YBX1 in C4-2 cells (Figure 1H,I, respectively). These results were validated using subcellular fractionation, which showed reduced nuclear YBX1 in AURKA-depleted cells (Figure 1J). 

To inspect whether AURKA regulates YBX1 in additional PCa cell lines, we examined subcellular localization of YBX1 in 22Rv1 cells ± either MLN8237 or AURKA shRNA. As observed in C4-2 cells, AURKA inhibition or depletion inhibited YBX1 nuclear translocation (Figure 1K,L). Subcellular fractionation of control and AURKA-depleted 22Rv1 cells further corroborated these findings (Figure 1M). Together, these results indicate that AURKA facilitates the nuclear residence of YBX1 in PCa cells.

### 2.3. AURKA Stabilizes YBX1 Protein, But Does Not Regulate Its mRNA Levels

**Figure 2:**
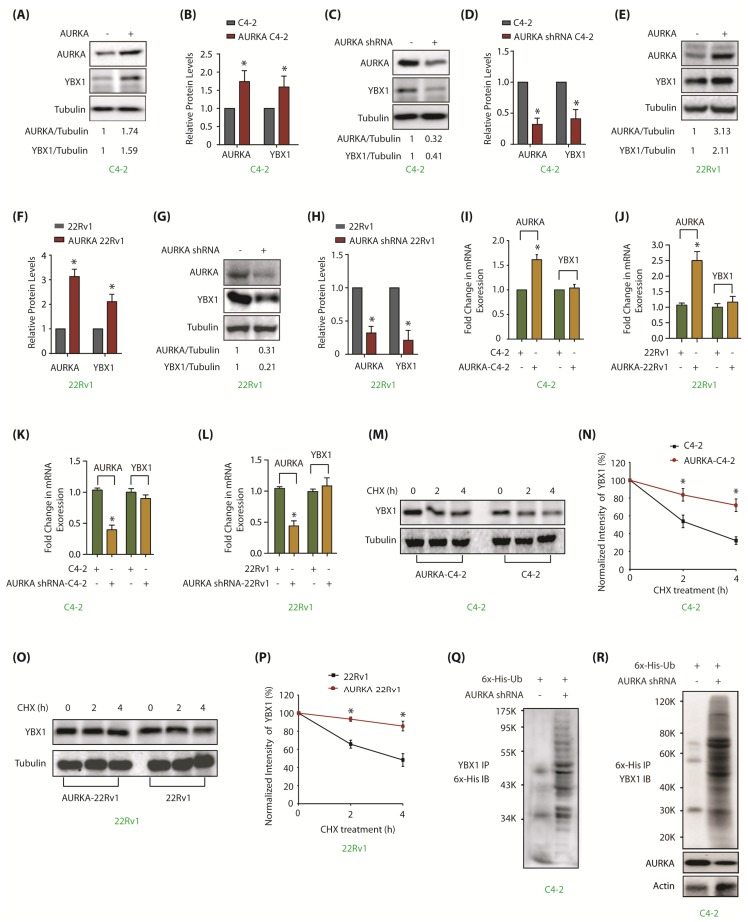
AURKA upregulates YBX1 levels by inhibiting its ubiquitylation in C4-2 and 22Rv1 cells. (**A**) Overexpression of AURKA increases YBX1 levels in C4-2 cells. (**B**) The data represents mean ± SEM of three independent experiments in C4-2 cells. * signifies significance at *p* < 0.05 compared to control. (**C**) AURKA ablation using AURKA shRNA depletes YBX1. C4-2 cells were exposed to control or AURKA shRNA for 30 h. (**D**) Histogram shows relative band intensities of AURKA and YBX1 normalized to the corresponding tubulin level from three independent experiments. (**E**) Overexpression of AURKA increases YBX1 levels in 22Rv1 cells. (**F**) Histogram shows relative band intensities of AURKA and YBX1 normalized to the corresponding tubulin level from three independent experiments. (**G**) AURKA ablation using AURKA shRNA (treated for 30 h) inhibits YBX1 protein levels in 22Rv1 cells. (**H**) Histogram shows relative band intensities of AURKA and YBX1 normalized to the corresponding tubulin level from three independent experiments. (**I**,**J**) AURKA does not regulate the mRNA levels of YBX1 in C4-2 and 22Rv1 cells, respectively. AURKA was stably overexpressed and YBX1 levels analyzed using real time qPCR. (**K**,**L**) AURKA does not regulate the mRNA levels of YBX1 in C4-2 and 22Rv1 cells, respectively. AURKA was knocked-down and YBX1 mRNA levels analyzed. C4-2 and 22Rv1 cells were exposed to control or AURKA shRNA for 30 h. (**M**) AURKA prevents YBX1 degradation. AURKA-C4-2 and C4-2 cells were treated with cycloheximide for 2 and 4 h, and YBX1 levels analyzed. (**N**) Graphical representation of YBX1 degradation rate. The YBX1 band intensity was normalized to tubulin and then normalized to the t=0 controls. Data are shown as mean± SEM from three different experiments (n = 3) * *p* < 0.05. (**O**) AURKA prevents YBX1 degradation in 22Rv1 cells. AURKA-22Rv1 and 22Rv1 cells were treated with cycloheximide for 2 and 4 h, and YBX1 levels analyzed. (**P**) Graphical representation of YBX1 degradation rate in 22Rv1 cells. The YBX1 band intensity was normalized to tubulin and then normalized to the t=0 controls. Data are shown as mean± SEM from three different experiments (n = 3) * *p* < 0.05. (**Q**) AURKA stabilizes YBX1 by inhibiting its ubiquitylation. 6x-His-Ubiquitin-expressing C4-2 cells were infected with either scrambled or AURKA shRNA lentivirus for 30 h and then treated with MG132 for 12 h. YBX1 was isolated and ubiquitylation analyzed using 6x-His antibody. (**R**) AURKA stabilizes YBX1 by inhibiting its ubiquitylation. Ubiquitylation was performed as described above, except ubiqitylated proteins were isolated using Ni-NTA beads followed by YBX1 IB. Each experiment was done at least three independent times and representative data are shown.

AURKA often regulates the protein stability of its substrates via phosphorylation [7,8,9,10], which prompted us to examine whether AURKA regulates YBX1. This was particularly important as YBX1 is upregulated in many cancers and shows strong correlation with disease recurrence, chemotherapy resistance and relapse after chemotherapy [20,28]. Ectopic overexpression of AURKA indeed increased YBX1 levels (Figure 2A). Figure 2B shows an average increase in YBX1 protein levels in AURKA-C4-2 cells from three independent experiments. Likewise, AURKA knockdown using corresponding shRNAs decreased YBX1 levels in C-42 cells (Figure 2C,D). Similar results were observed in 22Rv1 cells (Figure 2E–H), suggesting that AURKA positively controls YBX1 in PCa cells.

To uncover the molecular mechanism, we explored whether AURKA-triggered increase in YBX1 levels was at the transcriptional level. AURKA was overexpressed in C4-2 cells, which increased AURKA mRNA levels (>1.5 fold), but no change was observed in YBX1 mRNA levels (Figure 2I). Similar results were obtained in 22Rv1 cells (Figure 2J). To confirm these findings, AURKA was also knocked-down in C4-2 and 22Rv1 cells, however, YBX1 mRNA levels remain unaffected, suggesting that AURKA does not regulate YBX1 mRNA levels (Figure 2K,L).

As AURKA phosphorylates YBX1, we reasoned that AURKA likely regulates YBX1 post-translationally. We thus determined the half-life of YBX1 in cycloheximide-treated C4-2 and AURKA-C4-2 cells. AURKA overexpression increased YBX1 stability in both C4-2 (Figure 2M,N) and 22Rv1 cells (Figure 2O,P). As YBX1 degradation could be ubiquitin-dependent or independent mechanism, we expressed 6x-His-ubiquitin into C4-2 and AURKA-knocked-down-C4-2 cells, and evaluated YBX1 degradation. AURKA knockdown facilitated YBX1 ubiquitylation (Figure 2Q,R), thereby validating that AURKA stabilizes YBX1 levels by hindering its ubiquitylation (Supplementary Figure S1 includes raw data for Figure 2A,C,E,G,M,O). 

### 2.4. YBX1 Reciprocates and Positively Regulates AURKA Levels, But Not Its Subcellular Location 

**Figure 3:**
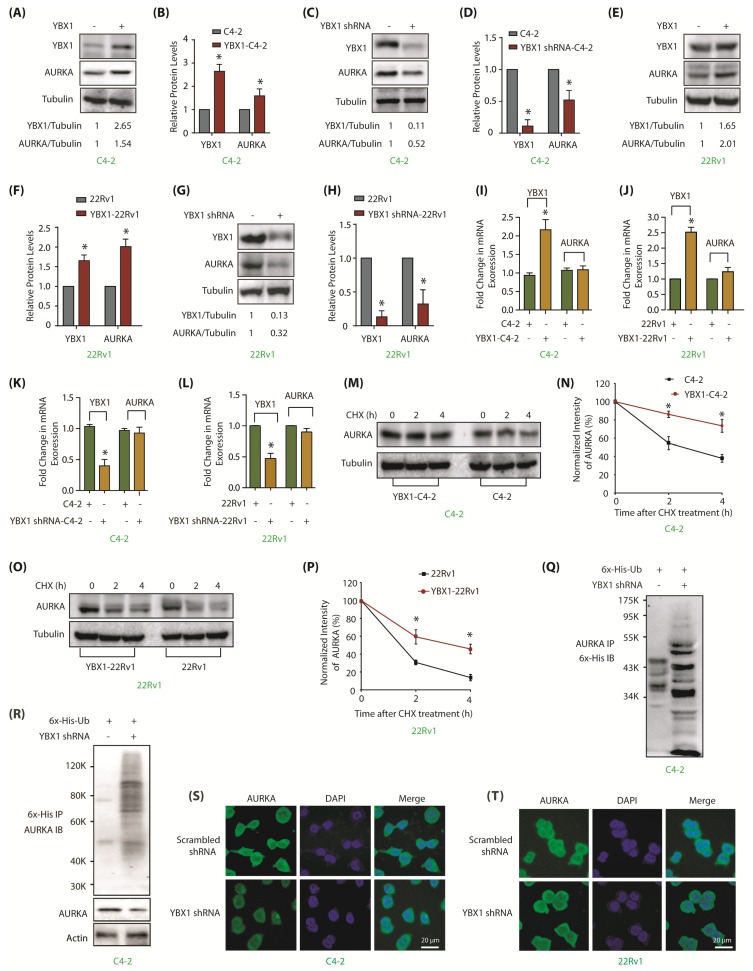
YBX1 stabilizes AURKA protein in C4-2 and 22Rv1 cells. (**A**) Overexpression of YBX1 enhances AURKA levels. (**B**) Histogram shows relative band intensities of YBX1 and AURKA normalized to the corresponding tubulin level from three independent experiments. * signifies significance at *p* < 0.05 compared to control. (**C**) YBX1 knock-down using YBX1 shRNA decreases AURKA in C4-2 cells. Cells were infected with control shRNA lentivirus (lane 1), or YBX1-shRNA lentivirus (lane 2), and AURKA and YBX1 levels were analyzed after 30 h. (**D**) Histogram shows relative band intensities of YBX1 and AURKA normalized to the corresponding tubulin level from three independent experiments. (**E**) YBX1 overexpression enhances AURKA levels in 22Rv1 cells. (**F**) Histogram shows relative band intensities of YBX1 and AURKA normalized to the corresponding tubulin level from three independent experiments. (**G**) YBX1 knockdown depletes AURKA protein levels in 22Rv1 cells. Cells were infected with control shRNA lentivirus (lane 1), or YBX1-shRNA lentivirus (lane 2), and AURKA and YBX1 levels were analyzed after 30 h. (**H**) Histogram showing relative band intensities of YBX1 and AURKA normalized to the corresponding tubulin level from three independent experiments. (**I**,**J**) YBX1 does not regulate the mRNA levels of AURKA in C4-2 and 22Rv1 cells, respectively. YBX1 was overexpressed and AURKA mRNA levels analyzed. (**K**,**L**) YBX1 does not regulate the mRNA levels of AURKA in C4-2 and 22Rv1 cells, respectively. YBX1 was knocked-down and AURKA mRNA levels analyzed. (**M**) YBX1 inhibits AURKA degradation. YBX1-C4-2 and C4-2 cells were treated with cycloheximide and AURKA levels evaluated. (**N**) Graphical representation of AURKA half-life in C4-2 cells. The results of densitometric scanning are shown with AURKA levels normalized to tubulin levels. (**O**) YBX1 prevents AURKA degradation in 22Rv1 cells. YBX1-22Rv1 and 22Rv1 cells were treated with cycloheximide and AURKA levels evaluated. (**P**) Graphical representation of YBX1 degradation rate in 22Rv1 cells. * *p* < 0.05. (**Q**) YBX1 stabilizes AURKA by inhibiting its ubiquitylation. 6x-His-Ubiquitin-expressing C4-2 cells were infected with either scrambled or YBX1 shRNA lentivirus for 30 h and then treated with MG132 for 12 h. AURKA was isolated and ubiquitylation analyzed using 6x-His antibody. (**R**) YBX1 stabilizes AURKA by inhibiting its ubiquitylation. Ubiquitylation was performed as described above, except that ubiqitylated proteins were isolated using Ni-NTA beads followed by AURKA IB. (**S**,**T**) YBX1 depletion does not impact the subcellular localization of AURKA in C4-2 and 22Rv1 cells, respectively. Cells were exposed to control or YBX1 shRNA for 30 h, fixed and stained with AURKA antibody (green) or DAPI (blue). >100 cells were analyzed from multiple random frames. Each experiment was done at least three independent times. Representative data are shown.

Many reports show that AURKA substrates reciprocally regulate AURKA levels [7,8,9,10]. Similar relationship was observed between YBX1 and AURKA, where YBX1 overexpression increased AURKA levels in C4-2 cells (Figure 3A). Figure 3B shows quantification of AURKA levels upon YBX1 overexpression from three independent experiments. We also depleted YBX1, which significantly decreased AURKA levels (Figure 3C,D). Comparable results were obtained in 22Rv1 cells, signifying the presence of AURKA-YBX1 reciprocal loop (Figure 3E–H). We further investigated whether YBX1-mediated regulation of AURKA was at mRNA level. YBX1 was overexpressed in C4-2 and 22Rv1 cells, which resulted in a 2–2.5 fold increase in its mRNA levels, nevertheless, AURKA mRNA levels remained unaltered, suggesting that YBX1 does not regulate AURKA at mRNA level (Figure 3I,J). Similar results were observed upon YBX1 knock-down in C4-2 and 22Rv1 cells, which displayed a minimal change in AURKA level, confirming that YBX1 does not increase the AURKA mRNA level (Figure 3K,L).

Further, we inspected AURKA and YBX1 levels in cycloheximide-treated C4-2 and YBX1-overexpressing C4-2 cells. YBX1 overexpression substantially reduced AURKA degradation, suggesting that YBX1 stabilizes AURKA protein (Figure 3M,N). Alike results were obtained in 22Rv1 cells (Figure 3O,P). To corroborate this observation, enhanced AURKA ubiquitylation was observed in YBX1-knocked down C4-2 cells as compare to control cells (Figure 3Q,R). Collectively, these results show that YBX1 stabilizes AURKA protein thus engaging in a positive reciprocal loop. Supplementary Figure S3 includes raw data for Figure 3A,C,E,G,M,O. 

We next explored whether YBX1 regulates AURKA subcellular residence. While AURKA was predominantly cytoplasmic, YBX1 showed both cytoplasmic and nuclear localization in both C4-2 and 22Rv1 cells (Figure 3S,T and Supplementary Figure S2) Notably, YBX1 depletion did not disturb AURKA localization in either cell type, suggesting that YBX1 does not regulate the subcellular localization of AURKA in these cells. 

### 2.5. AURKA Regulates YBX1 Levels and Subcellular Localization via Phosphorylation

**Figure 4:**
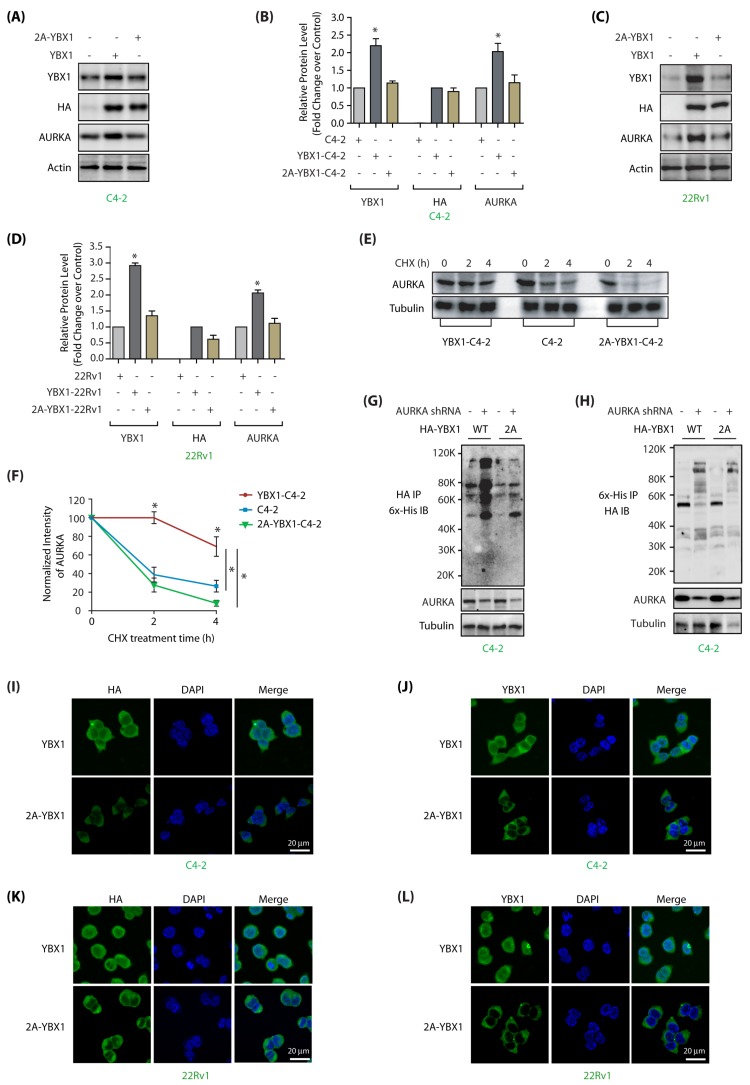
AURKA regulates stability and subcellular localization of YBX1 by phosphorylation in C4-2 and 22Rv1 cells. (**A**) AURKA increases YBX1 levels by phosphorylation. C4-2 cells were infected with HA-tagged wild-type YBX1 or 2A-YBX1 retrovirus. After 30 h, protein levels were analyzed using YBX1, AURKA, HA and actin antibodies. (**B**) Graphical representation of YBX1 and AURKA levels in C4-2 cells expressing either wild-type or mutant YBX1. Average values of wild-type and mutant YBX1 levels from three independent experiments are illustrated in the graph. The statistical significance was analyzed using one way ANOVA, * *p* < 0.05, compared to control. (**C**) AURKA increases YBX1 levels by phosphorylation in 22Rv1 cells, as 2A-YBX1 showed much less steady state levels. (**D**) Graphical representation of YBX1 and AURKA levels in 22Rv1 cells expressing either wild type or mutant YBX1 from three independent experiments. (**E**) AURKA increases the stability of YBX1 via phosphorylation. AURKA levels were analyzed in C4-2, YBX1-C4-2 and 2A-YBX1-C4-2 cells treated with cycloheximide. (**F**) Graphical representation of AURKA half-life in C4-2, YBX1-C4-2 and 2A-YBX1-C4-2 cells. (**G**) AURKA depletion increased the ubiquitylation of WT YBX1, but not 2A-YBX1 in C4-2 cells. 6x-His-Ubiquitin expressing WT YBX1-C4-2 and 2A-YBX1-C4-2 cells were treated with either scrambled or AURKA shRNA lentivirus for 30 h, followed by MG132 treatment for 12 h. YBX1 was isolated using HA antibody and ubiquitylation analyzed using 6x-His antibody. (**H**) AURKA depletion increased the ubiquitylation of WT YBX1, but not 2A-YBX1 in C4-2 cells. Ubiquitylation was performed as described above, except ubiqitylated proteins were isolated using Ni-NTA beads followed by HA IB. (**I**,**J**) AURKA regulates the subcellular localization of YBX1 via phosphorylation. HA-tagged wild-type and 2A-YBX1 expressing cells were stained using DAPI (blue), HA antibody (green in 4I) or YBX1 antibody (green in 4J). More than 100 cells were analyzed from multiple frames. Representative data are shown (>95%). (**K**,**L**) AURKA controls YBX1 subcellular localization via phosphorylation in 22Rv1 cells (analyzed using HA antibody in 4K and YBX1 in 4L).

We next examined whether AURKA increases YBX1 stability via phosphorylation. Wild type YBX1 and 2A-YBX1 were ectopically expressed in C4-2 cells, which showed high level of the former, but considerably lower level of the latter, indicating that AURKA stabilizes YBX1 by using phosphorylation (Figure 4A; Supplementary Figure S3A shows full gel pictures of Figure 4A). Besides, as 2A-YBX1 was present in lower level, it caused a simultaneous attenuation in AURKA level, apparently due to the reciprocal cross-talk. Figure 4B represents relative protein levels of YBX1 and 2A-YBX1 in C4-2 cells from three independent experiments. Analogous consequences were observed in 22Rv1 cells, indicating the reciprocal cross-talk between YBX1 and AURKA may be a common mechanism in PCa cells (Figure 4C,D and Supplementary Figure S3C).

We further treated C4-2, and YBX1 and 2A-YBX1-expressing cells with cycloheximide to inhibit protein synthesis, and analyzed the half-life of AURKA. WT-YBX1 expressing cells showed highest stability of AURKA, likely due to the concomitant increase in YBX1 level, which stabilizes AURKA. By contrast, 2A-YBX1 cells showed rapid degradation of AURKA, seemingly due to less YBX1, as well as increased ubiquitylation (Figure 4E,F; Supplementary Figure S3E).

To examine this premise, we compared the ubiquitylation of ectopically expressed YBX1 in YBX1-C4-2 and 2A-YBX1-C4-2 cells using HA antibody with and without transient AURKA knockdown. AURKA depletion showed enhanced ubiquitylation of wild-type YBX1 as compared to 2A-YBX1, thereby indicating that AURKA increases YBX1 stability by phosphorylation (Figure 4G,H).

To determine whether AURKA mediates YBX1 nuclear translocation by phosphorylating it, we examined the localization of HA-tagged wild-type and mutant 2A-YBX1 in C4-2 cells using YBX1 and HA antibodies. Ectopically expressed wild-type YBX1 was both cytoplasmic and nuclear localized similar to endogenous enzyme, but 2A mutant was predominantly cytoplasmic indicating that AURKA promotes nuclear residence of YBX1 by phosphorylation (Figure 4I,J). 22Rv1 cells revealed similar regulation of WT and mutant YBX1 (Figure 4K,L). Together, these results support that AURKA facilitates nuclear translocation of YBX1 by phosphorylation.

### 2.6. AURKA and YBX1 Feedback Circle Fuels Aggressive Phenotypes Including EMT and CSC

**Figure 5:**
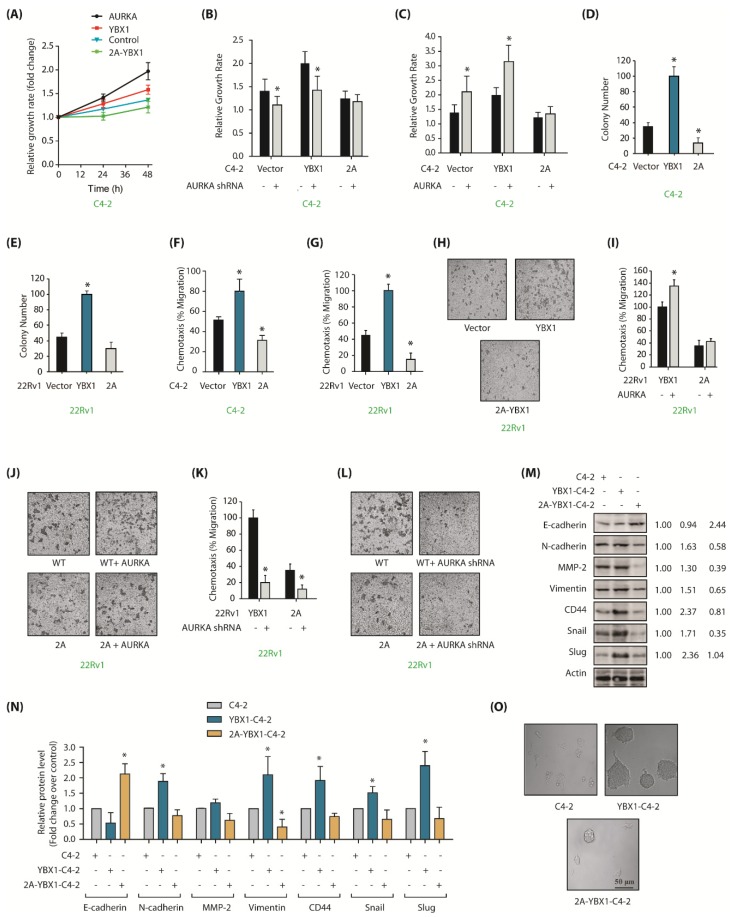
YBX1 is an important cancer target of AURKA in CRPC cells. (**A**) YBX1 stimulates cell proliferation in C4-2 cells. C4-2, AURKA-C4-2, YBX1-C4-2 and 2A-YBX1-C4-2 cells were grown for 24 and 48 h, followed by an MTT assay. (**B**) AURKA knockdown decreases proliferation in C4-2 and YBX1-C4-2 cells, but not in 2A-C4-2 cells. C4-2, YBX1-C4-2 and 2A-YBX1-C4-2 cells were exposed to either scrambled shRNA or AURKA shRNA lentivirus for 48 h, followed by an MTT assay. (**C**) AURKA overexpression increases cell proliferation in C4-2 and YBX1-C4-2 cells, but not in 2A-C4-2 cells. C4-2, YBX1-C4-2 and 2A-C4-2 cells were exposed to either vector or AURKA retrovirus for 48 h, followed by an MTT assay. (**D**) Overexpression of WT-YBX1 shows higher colony formation compared to 2A-C4-2 cells using a soft agar assay. (**E**) Overexpression of WT-YBX1 shows higher colony formation in 22Rv1 cells compared to 2A-22Rv1 cells. (**F**) YBX1 promotes cell migration in C4-2, whereas 2A-YBX1 acts as dominant negative. Histogram shows mean ± SEM of three independent experiments. * *p* < 0.05 compared to vector-expressing control. (**G**) YBX1 promotes cell motility in 22Rv1 cells, whereas 2A-YBX1 acts as dominant negative. Histogram shows mean ± SEM of three independent experiments. * *p* < 0.05 compared to vector-expressing control. (**H**) YBX1 promotes cell motility in 22Rv1 cells. Representative pictures are shown. (**I**) AURKA overexpression accelerates proliferation in YBX1-22Rv1 cells, however not in 2A-YBX1-22Rv1 cells. Histogram shows mean ± SEM of three independent experiments. * *p* < 0.05 compared to vector-expressing control. (**J**) Representative pictures showing that AURKA overexpression accelerates proliferation in YBX1-22Rv1 cells, but not in 2A-YBX1-22Rv1 cells. These experiments were performed three independent times and representative pictures are shown. (**K**) AURKA knockdown inhibits cell motility in both YBX1-22Rv1 and 2A-YBX1-22Rv1 cells. C4-2, YBX1-C4-2 and 2A-C4-2 cells were exposed to either scrambled shRNA or AURKA shRNA lentivirus for 48 h, followed by chemotaxis assay. (**L**) Representative pictures showing that AURKA depletion decreases cell motility in both YBX1-22Rv1 and 2A-YBX1 22Rv1 cells. Magnification, 200×. (**M**) AURKA-mediated phosphorylation of YBX1 facilitates EMT. Ectopic expression of YBX1 increases EMT and CSC proteins, but decreases E-cadherin. 2A-YBX1 decreases EMT and CSC proteins but increases E-cadherin levels. (**N**) Histograms showing relative levels of EMT markers and E-cadherin from three independent experiments in C4-2, YBX1-C4-2 and 2A-YBX1-C4-2 cells. (**O**) YBX1 overexpression increases sphere-forming ability in C4-2 cells.

YBX1 upregulation is associated with poor prognosis in PCa [28]. Therefore, we assessed the contribution of AURKA-YBX1 cross talk in enabling aggressive phenotypes in PCa cells. AURKA or YBX1 overexpression caused enhanced proliferation in C4-2 cells as predicted (Figure 5A). By contrast, 2A-YBX1 expression significantly impaired proliferation rate, which was considerably less than parental C4-2 cells (Figure 5A). Further, AURKA knockdown diminished proliferation in YBX1-C4-2 cells, but not in mutant expressing cells, signifying that the YBX1-triggered increase in cellular proliferation is mostly caused by AURKA (Figure 5B). We further confirmed this observation by ectopically expressing AURKA, which led to enhanced proliferation in YBX1-C4-2 cells, but not in mutant expressing cells. These findings suggest that AURKA facilitates cell growth by phosphorylating YBX1 (Figure 5C). 

The impact of YBX1 phosphorylation by AURKA was next evaluated under anchorage-independent conditions. YBX1-expressing cells showed significantly higher number of colonies as compared to vector-expressing C4-2 cells in the soft agar assay, whereas 2A-YBX1-C4-2 cells showed much fewer colonies (Figure 5D). We observed similar results in 22Rv1, YBX1-22Rv1 and 2A-YBX1-22Rv1 cells (Figure 5E). These findings show that YBX1 phosphorylation by AURKA facilitates proliferation in both adherent-dependent and adherent-independent situations in PCa cells. 

Further, a strong enhancement in cell migration was noted upon YBX1 expression, but not upon 2A-YBX1 expression, demonstrating that AURKA facilitates cell migration by phosphorylating YBX1 (Figure 5F). 22Rv1 cells showed similar regulation (Figure 5G,H). To further explore AURKA’s role in this process, we overexpressed AURKA in YBX1-22Rv1 and 2A-YBX1-22Rv1 cells, which substantially augmented cell migration in WT YBX1 cells, but not in mutant expressing cells (Figure 5I,J). We further depleted AURKA in YBX1-22Rv1 and 2A-YBX1-22Rv1 cells, which surprisingly decreased cell migration in both wild-type YBX1 and 2A-YBX1-expressing cells, suggesting that AURKA promotes cell motility by phosphorylating other targets as well (Figure 5K,L). Nevertheless, these results corroborate a lethal role of AURKA-YBX1 synergistic crosstalk in CRPC. 

As YBX1 stimulates EMT [23], we investigated whether AURKA-mediated YBX1 phosphorylation promotes EMT. We examined the protein levels of several EMT-inducing proteins including Snail, CD44, matrix metalloproteinase-2 (MMP2), N-cadherin, vimentin and Slug in WT and 2A-expressing cells. C4-2 cells were used as controls. We also investigated the levels of E-cadherin, which decreases during the EMT process. All EMT-promoting proteins increased considerably upon YBX1 expression, but 2A-YBX1 performed as dominant-negative and fully repressed their expression (Figure 5M). Furthermore as expected, YBX1 decreased E-cadherin levels but 2A-YBX1 expression increased it. Figure 5N shows the average levels of epithelial, CSC and EMT proteins from three independent experiments. 

We next determined the CSC-forming potential of these cells using sphere forming assay. YBX1 expression induced huge prostatotosphere formation, whereas neither C4-2 nor 2A-YBX1C4-2 cells showed any prostatosphere (Figure 5O). These data thus support the model that AURKA-YBX1 crosstalk significantly contributes to EMT and CSC phenotypes.

### 2.7. AURKA-Mediated YBX1 Phosphorylation Differentially Regulates AR and ARV7 mRNA and Proteins 

Shoita et al. previously showed that YBX1 binds to AR promoter and increases its transcription in LNCaP and 22Rv1 cells [21], however, later studies from the same group revealed that YBX1 increases *ARv7* mRNA but not *AR* mRNA levels [22]. Thus, we investigated whether wild type or mutant YBX1 modulates *AR* and *ARv7* mRNA levels in C4-2 and 22Rv1 cells. 

**Figure 6:**
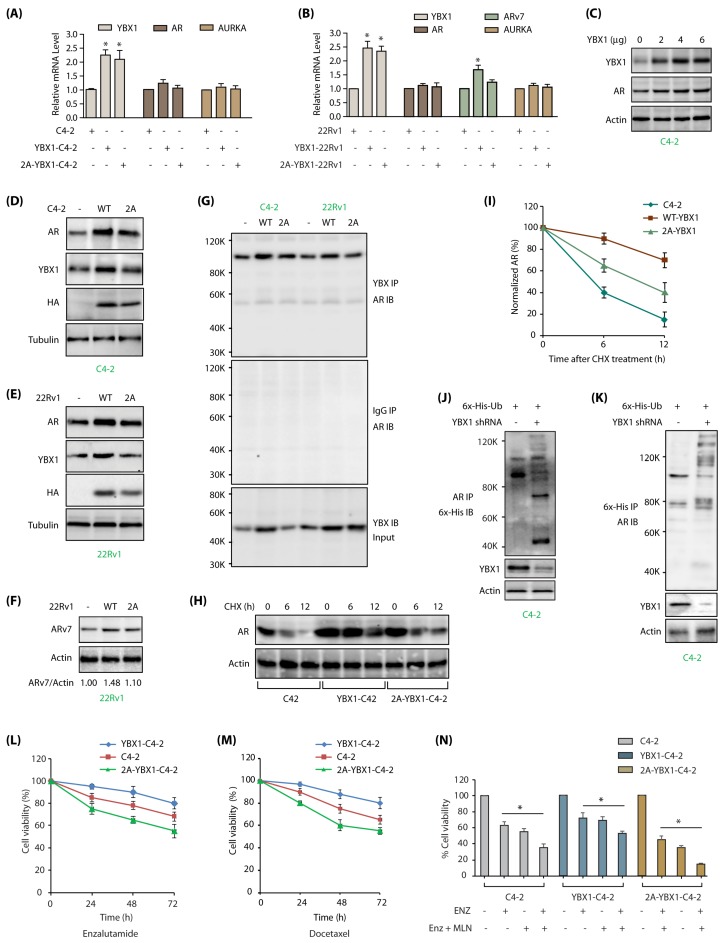
YBX1-mediated regulation of AR and ARv7 in C4-2 cells. (**A**) YBX1 does not regulate *AR* or *AURKA* mRNA levels in C4-2 cells. *YBX1, AR* and *AURKA* mRNA levels were analyzed as described in Materials and Methods in C4-2, WT YBX1-C4-2 and 2A-YBX1-C4-2 cells. (**B**) YBX1 upregulation increases *ARv7* mRNA levels in 22Rv1 cells. *YBX1, AR, ARv7* and *AURKA* mRNA levels were analyzed in 22Rv1, WT YBX1-22Rv1 and 2A-YBX1-22Rv1 cells. (**C**) AR levels positively correlates with YBX1 levels in transfected C4-2 cells. Different amounts of YBX1 DNA was transfected in C4-2 cells. YBX1 and AR levels were analyzed after 30 h. (**D**) AR levels positively correlates with YBX1 levels in vector, YBX1 and 2A-YBX1-expressing C4-2 cells. (**E**) AR levels positively correlates with YBX1 levels in vector, YBX1 and 2A-YBX1-expressing 22Rv1 cells. (**F**) ARv7 levels positively correlates with YBX1 levels in vector, YBX1 and 2A-YBX1-expressing 22Rv1 cells. (**G**) AR binds YBX1 in vector, YBX1 and 2A-YBX1-expressing C4-2 and 22Rv1 cells. YBX1 was immunoprecipitated from vector, YBX1 and 2A-YBX1-expressing C4-2 and 22Rv1 cells, followed by AR immunoblotting (top panel). Middle panel shows IgG IP in the same six cell lines, followed by AR IB. Bottom panel shows YBX1 levels in the input. (**H**) YBX1 prevents AR degradation. AR levels were analyzed in C4-2, YBX1-C4-2 and 2A-YBX1-C4-2 cells following treatment with cycloheximide for 6 and 12 h. (**I**) Graphical representation of AR half-life. The results of densitometric scanning are shown graphically with YBX1 signal normalized to actin signal. * *p* < 0.05. (**J**) YBX1 stabilizes AR by inhibiting its ubiquitylation. 6x-His-Ubiquitin expressing C4-2 cells were treated with either scrambled or YBX1 shRNA lentivirus for 30 h, followed by MG132 treatment for 12 h. AR was isolated and ubiquitylation analyzed using 6x-His antibody. (**K**) YBX1 stabilizes AR by inhibiting its ubiquitylation. Ubiquitylation was performed as described above, except ubiqitylated proteins were isolated using Ni-NTA beads followed by AR IB. Each experiment was done at least three independent times. Representative data are shown. (**L**) AURKA-mediated phosphorylation of YBX1 promotes enzalutamide-resistance. C4-2, YBX1-C4-2 and 2A-YBX1-C4-2 cells were plated overnight, enzalutamide (1 µM) was added and cells grown for additional 24, 48 or 72 h, followed by MTT assay. (**M**) YBX1 overexpression increases docetaxel resistance in C4-2 cells. C4-2, YBX1-C4-2 and 2A-YBX1-C4-2 cells were plated overnight, followed by docetaxel (100 nM) treatment. The cells were grown for additional 24, 48 or 72 h, followed by MTT assay. (**N**) 2A-YBX1 overexpression sensitizes C4-2 cells to AURKA inhibitor MLN8237 and enzalutamide, whereas YBX1 overexpression confers resistance. C4-2, YBX1-C4-2 and 2A-YBX1-C4-2 cells were plated overnight, followed by enzalutamide (1 µM) or MLN8237 (1 µM) treatments either independently or in combination. The cells were grown for additional 48 h, followed by MTT assay.

We overexpressed wild-type and 2A-YBX1 in C4-2 cells, both of which showed >2 fold increase in their mRNA levels. However, *AR* mRNA levels were not impacted by YBX1 overexpression, indicating that YBX1 does not regulate *AR* in C4-2 cells (Figure 6A). We also investigated *AURKA* mRNA levels, which remained unaltered upon YBX1 overexpression, as we observed previously (Figure 3I). 

We examined the effect of YBX1 overexpression in 22Rv1 cells, which express both AR and ARv7. Overexpression of YBX1 and 2A-YBX1 resulted in >2 fold increase in their mRNA levels (Figure 6B). However, similar to C4-2 cells, *AR* mRNA levels did not alter in either case. In contrast, we observed ~1.5 fold increase in *ARv7* mRNA levels upon YBX1 overexpression, but not in 2A-YBX1 expressing cells, signifying that YBX1 phosphorylation by AURKA contributes to ARv7 signaling in CRPC (Figure 6B). *AURKA* mRNA remained unaffected by either YBX1 or 2A-YBX1 overexpression, confirming that YBX1 regulates AURKA at the protein level only in PCa cells.

YBX1 has not been linked to AR stability. To examine whether YBX1 regulates AR at the protein level, we initially transfected different amounts of YBX1 DNA in C4-2 cells, and analyzed AR levels, which revealed a positive correlation between YBX1 and AR levels (Figure 6C). This finding was confirmed in YBX1 and 2A-YBX1-expressing C4-2 and 22Rv1 cells, both of which showed an increase in AR protein upon WT-YBX1 expression, compared to 2A-mutant (Figure 6D,E, respectively). Further, ARv7 protein level also increased in 22Rv1 cells upon YBX1 overexpression (Figure 6F; Supplementary Figure S4F shows raw data for 6F). To investigate whether YBX1 regulates AR post-translationally, as the first step, we analyzed potential association of YBX1 and AR. YBX1 associates with AR in both C4-2 and 22Rv1 cells (Figure 6G). This finding prompted us to determine the stability of AR in C4-2, YBX1-C4-2 and 2A-YBX1-C4-2 cells. AR displayed higher stability in the presence of YBX1, indicating that YBX1 presumably stabilizes AR protein (Figure 6H,I; Supplementary Figure S4H includes raw data of Figure 6H). YBX1 knockdown indeed led to increased ubiquitylation of AR, thereby confirming that YBX1 increases AR levels by preventing its degradation (Figure 6J,K).

### 2.8. AURKA-Mediated YBX1 Phosphorylation Increases Resistance to Enzalutamide and Docetaxel 

Inhibition of YBX1 pathway confers enzalutamide sensitivity [22,29]. Therefore, we tested enzalutamide-sensitivity in C4-2 cells, which showed ~75% cell viability in 72 h (Figure 6L). Wild-type YBX1 expression in C4-2 cells conferred enzalutamide resistance as expected (>80% cell viability), whereas 2A-YBX1 expression sensitized them to enzalutamide-induced toxicity (~55% cell viability) (Figure 6L). 

We next investigated whether YBX1 phosphorylation by AURKA contributes to docetaxel-resistance. AURKA inhibition using alisertib (aka MLN8237) sensitizes DU145 cells to docetaxel [30], suggesting that YBX1 phosphorylation may contribute to drug resistance. C4-2, YBX1-C4-2 and 2A-YBX1-C4-2 cells were exposed to docetaxel (100 nM), which showed that WT YBX1 overexpression confers docetaxel resistance, but 2A-YBX1 mutant sensitizes it (Figure 6M). These results indicate that YBX1 phosphorylation by AURKA is critical for inducing chemoresistance. These findings were further confirmed using alisertib in combination with enzalutamide in C4-2, YBX1-C4-2 and 2A-YBX1-C4-2 cells. As shown in Figure 6N, 2A-YBX1 cells are highly sensitive to the combination therapy, compared to C4-2 and YBX1-C4-2, underlining the significance of AURKA-YBX1 loop in promoting chemoresistance in C4-2 cells. 

### 2.9. AURKA-Mediated YBX1 Phosphorylation Promotes Tumorigenesis In Vivo

**Figure 7:**
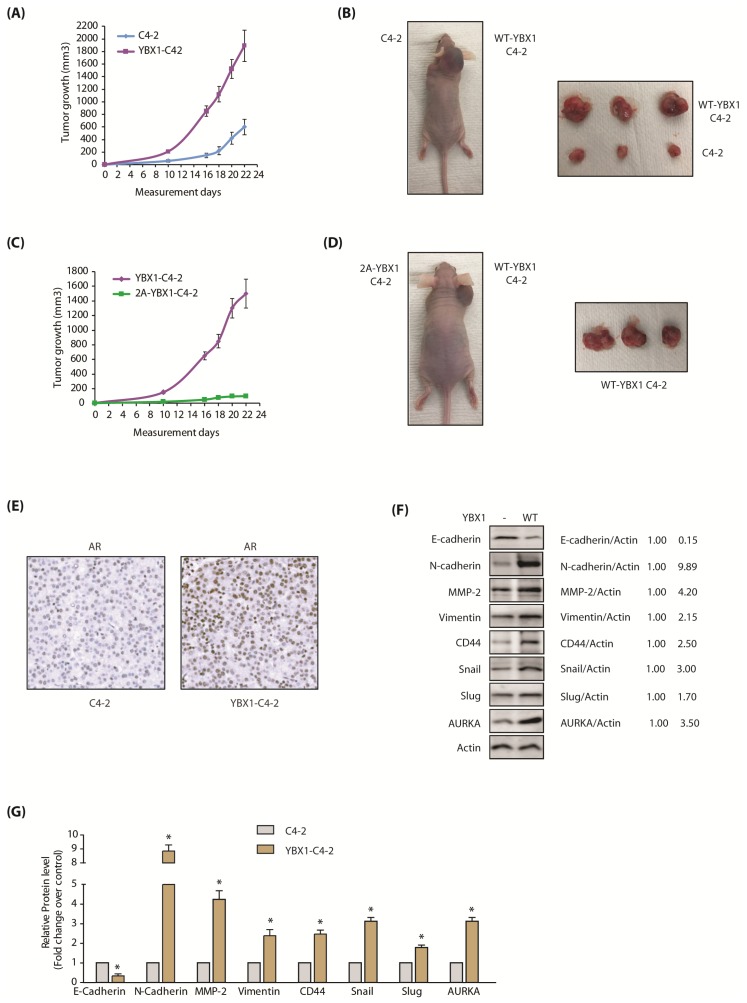
The AURKA-YBX1 loop promotes tumorigenesis in vivo. (**A**) YBX1 overexpression enhances tumorigenesis in vivo. Three male nude athymic mice were injected with C4-2 cells and YBX1-C4-2 cells on left and right shoulders, respectively. (**B**) Nude athymic mice were inoculated with YBX1-C4-2 cells and C4-2 cells on the right and left shoulders, respectively. The pictures were taken 22 days following injection. Representative images are shown. (**C**) 2A-YBX1 expression inhibits tumor growth in vivo. Three nude mice were injected with 2A-YBX1-C4-2 and YBX1-C4-2 cells on the left and right shoulders, respectively. (**D**) Athymic nude mouse were inoculated with YBX1-C4-2 cells and 2A-YBX1-C4-2 cells on right and left shoulders, respectively. The pictures were taken 22 days following inoculation. Representative images are shown. (**E**) YBX1 upregulates AR levels in vivo. AR immunohistochemistry in C4-2 and YBX1-C4-2 xenografts. (**F**) Expression levels of AURKA, EMT proteins, E-cadherin and CSC proteins in control C4-2 and YBX1-C4-2 xenografts. Following euthanization, tumor tissues were isolated and flash-frozen in liquid nitrogen. Tumor tissues were homogenized and expression levels of various EMT proteins, E-Cadherin and AURKA analyzed. (**G**) Histogram shows relative band intensities normalized to the corresponding actin level. Data shown as mean ± SEM of three independent experiments. * signifies significance at *p* < 0.05 compared to control.

The consequences of YBX1 phosphorylation by AURKA in facilitating tumorigenesis was next analyzed in vivo. C4-2 and YBX1-C4-2 cells were subcutaneously inoculated in athymic nude mice on the left and right shoulders, respectively. YBX1-C4-2 cells formed significantly bigger tumors, compared to C4-2 cells (Figure 7A,B). In another set of experiments, YBX1-C4-2 cells and 2A-YBX1-C4-2 were subcutaneously injected on right and left shoulders, respectively, of athymic nude mice. The tumors were quantifiable after 8 days. As anticipated, YBX1-C4-2 cells formed large tumors and 2A-YBX1-C4-2 cells showed negligible tumors (Figure 7C,D). These results validate that YBX1 phosphorylation by AURKA contributes significantly to AURKA-mediated tumorigenesis in vivo.

### 2.10. YBX1 Expression Increases the Levels of AR and EMT-Inducing Proteins in C4-2 Xenografts 

Our data showed that YBX1 stabilizes AR protein (Figure 6H–K), therefore, we examined whether this phenomenon occurs in vivo. YBX1 levels were analyzed in C4-2 and YBX1-C4-2 xenografts, which revealed that AR is indeed increased in YBX1-expressing xenografts (Figure 7E; Supplementary Figure S5E includes raw data for each panel), indicating that YBX1 positively regulates AR protein in vivo as well. 

**Figure 8:**
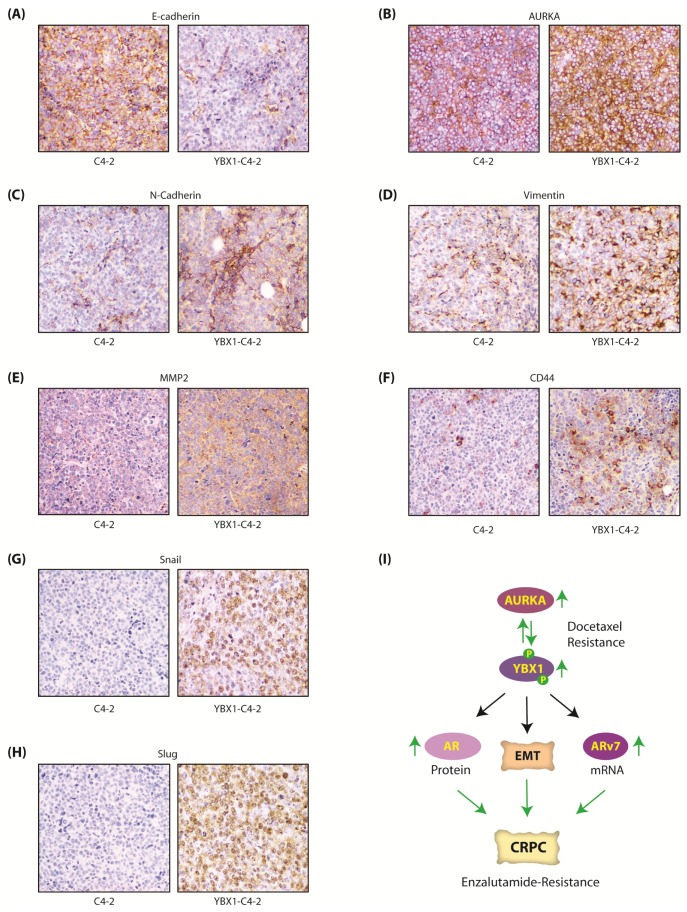
The AURKA-YBX1 feedback loop facilitates EMT in vivo. (**A**) E-Cadherin (**B**) AURKA (**C**) N-Cadherin (**D**) Vimentin (**E**) MMP2 (**F**) CD44 (**G**) Snail and (**H**) Slug proteins in C4-2 and YBX1-C4-2 xenografts as determined by the immunohistochemistry (IHC) assay. (**I**) Proposed model showing the consequences of AURKA-YBX1 synergy in CRPC pathogenesis.

We also explored the levels of EMT-inducing proteins in C4-2 and YBX1-C4-2 xenografts using both immunoblotting and immunohistochemistry. As 2A-YBX1-C4-2 cells shows no tumor formation, they could not be analyzed. In agreement with our cellular data, E-cadherin significantly decreased in YBX1-C4-2 xenografts, compared to C4-2 tumors (Figure 7F). Similarly, all EMT markers increased significantly in YBX1 overexpressing tumors, compared to parental C4-2 tumors (Figure 7F,G). Finally, AURKA was considerably higher in YBX1 overexpressing tumors, supporting YBX1-AURKA reciprocal loop in vivo. The IHC analysis confirmed these findings in vivo, which showed less E-cadherin (Figure 8A), but significantly high levels of AURKA and all EMT-inducing proteins (Figure 8B–H). These data further support that YBX1 promotes EMT in CRPC in vivo. 

## 3. Discussion

YBX1 is a transcription factor, which is mainly cytoplasmic, but it shuttles to the nucleus upon noxious stimuli such as UV irradiation, hyperthermia and chemotherapeutic drugs [20,31]. YBX1 has vital roles in both cytoplasm and nucleus and regulates multiple biological events including pre-mRNA splicing, chromatin remodeling, transcription and translation. YBX1 is overexpressed in multiple cancers and regulates cell proliferation and survival, DNA replication and repair, multi-drug resistance and EMT [32,33]. In PCa patients, high nuclear and cytoplasmic YBX1 expression strongly correlates with poor prognosis [34].

YBX1 is shown to be phosphorylated at several sites including S102, S165 and S176. The most well characterized site is S102 on YBX1, which is phosphorylated by Akt and p90Rsk causing its activation and tumorigenesis [35,36,37]. YBX1 is phosphorylated at S165 and S176 in colon cancer, which promotes NF-κB activation and tumorigenic potential, however, the kinase responsible for these phosphorylation events was not conclusively identified [38,39]. While all these phosphorylation events results in increased nuclear translocation of YBX1, to date no study has shown that YBX1 is stabilized by phosphorylation. This is particularly important as YBX1 protein abundance, but not its DNA status or mRNA levels, is predictive of PCa recurrence [40], suggesting that YBX1 is upregulated predominantly due to increased stabilization in PCa. Thus, targeting YBX1 stabilization may be an alternative approach to prevent tumor relapse. 

We uncovered that AURKA directly phosphorylates YBX1 at S62 and S102, which results in increased protein stability leading to aggressive malignant phenotypes including EMT, CSC and chemoresistance. YBX1 reciprocates and stabilizes AURKA by hindering its ubiquitylation, thereby initiating a synergistic cross-talk. Significantly, phospho-dead YBX1 is dominant negative, and decreases the basal levels of EMT-inducing proteins in C4-2 cells, underlining a fatal role of AURKA in YBX1-mediated malignancy. 

Active AR signaling is a critical driver for castration-resistance progression of PCa. Yet, ~20–40% CRPC patients exhibit primary resistance to enzalutamide, and patients that originally are sensitive to enzalutamide later attain secondary resistance, both of which are incurable. Primary enzalutamide resistance often arises due to ligand-independent AR spliced isoforms such as ARv7, which is constitutively active [22]. Our study uncovered that YBX1 increases *ARv7* mRNA levels, but not *AR* mRNA levels. Instead it upregulates AR post-translationally by preventing its ubiquitylation. This finding was also confirmed in vivo, which showed higher YBX1 protein in YBX1-C4-2 xenografts compared to C4-2 xenografts. While YBX1 is linked to increased transcription of AR and ARv7, it has not been investigated in regulating AR stability. As AR signaling is critical for both androgen-dependent and castration-resistant PCa, this study highlights the clinical implication of YBX1-AURKA synergy in PCa progression (Figure 8I). 

## 4. Materials and Methods 

### 4.1. Cell Lines and Antibodies

C4-2, HEK293, Phoenix and 22Rv1 cell lines were purchased from ATCC. AURKA, Actin and YBX1 antibodies were bought from Santa Cruz Biotech (Santa Cruz, CA, USA). N-cadherin, CD44, Slug and Snail antibodies were bought from One World Lab (San Diego, CA, USA). E-cadherin, MMP-2 and Vimentin antibodies were obtained from Bioss (Woburn, MA, USA). All validated antibodies were used at 1-1000 dilution. Details of antibodies are provided in Supplementary Table S1. 

### 4.2. YBX1 and AURKA shRNAs

AURKA shRNAs in pLKO vector were generated in our preceding study [7]. Human YBX1 shRNA was cloned in pLKO.1 vector [41]. The sequences of YBX1 shRNAs are as follows: *YBX1 shRNA (forward)* CCG GGA GAA CCC TAA ACC ACA AGA TCT CGA GAT CTT GTG GTT TAG GGT TCT CTT TTT G. *YBX1 shRNA (reverse)* AAT TCA AAA AGA GAA CCC TAA ACC ACA AGA TCT CGA GAT CTT GTG GTT TAG GGT TCT C. Scrambled, AURKA and YBX1 shRNA lentiviruses were used to infect PCa cells. Stable cell lines were generated by puromycin selection.

### 4.3. In Vitro Kinase Assays

In vitro phosphorylations of 6x-His-tagged wild-type or mutant YBX1) were conducted using recombinant AURKA-TPX2 complex and 0.5 μCi of [γ-^32^P]ATP in kinase buffer (50 mM Tris, 10 mM MgCl_2_) for 30 min. The proteins were separated by SDS-PAGE gel, transferred and exposed for autoradiography.

### 4.4. Expression Plasmids, Expression and Purification of AURKA, TPX2 and YBX1

HA-tagged YBX1 was cloned at BamHI and Xho1 sites in VIP3 and TAT-HA vectors. HA-tagged YBX1 mutants were created using site-directed overlap PCR. AURKA was generated in insect cells as reported before [7]. 6x-His-tagged TPX2 and 6x-His-tagged wild-type and mutant YBX1 were generated in *E. coli* and isolated as we described before [42]. AURKA and YBX1 retroviruses were used to infect PCa cells as described before [43]. 

### 4.5. Ubiquitylation Assay

6x-His–ubiquitin expressing C4-2 cells were infected with YBX1 or AURKA shRNA lentivirus for 30h. Next 10 μM MG132 (Sigma) was added for another 12 h. Following cell lysis, ubiquitylated proteins were purified using Ni-NTA beads. The proteins were separated, transferred to PVDF membrane and detected using AURKA, HA or YBX1 antibodies. Alternatively, ubiquitylated proteins were purified using AURKA, HA or YBX1 antibodies, and analyzed using 6x-His antibody.

### 4.6. Chemotaxis Assay

Cell migration assays were performed in triplicates, four independent times using Boyden chambers [44]. The data was normalized when comparing multiple assays, and expressed as a percentage cell number present on the membrane.

### 4.7. MTT Assay

MTT assay was performed as we reported before [45]. Experiments were repeated three independent times in triplicate.

### 4.8. Immunofluorescence

PCa cells grown on poly-l-lysine coated coverslips were fixed using 4% formaldehyde, then washed thrice using PBS. They were next incubated in blocking buffer (1%FBS, 2% BSA, 0.1% triton X-100 in PBS) for 1 h. Cells were incubated with AURKA or YBX1 antibodies for 3 h, followed by fluorescein-isothiocyanate-conjugated secondary antibody. Cells were pictured using Nikon Eclipse E600 microscope (Nikon Instruments, Melville, NY, USA).

### 4.9. Real-Time qPCR

The details are included in our previous study [46]. Primer sequences are presented in Supplementary Table S2.

### 4.10. Soft agar Colony Formation 

Soft agar assay was conducted as described in our previous study [9,10].

### 4.11. Prostatosphere Assay

Prostatosphere assay was conducted as described before [9,10]. 

### 4.12. In Vivo Xenograft in Nude Mice

All animal experiments were done in accordance with institutional guidelines of Purdue University (*Purdue Animal Care and Use Committee* aka PACUC) using an approved protocol # 1111000292 (approval date 01/07/2019). Animal care was in accordance with institution guidelines. Male athymic nude mice 4 weeks of age were obtained from Taconic Laboratories and injected as before [8]. Tumor bearing mice exhibited no weight loss compared with control mice. The animals were euthanized 22 days after tumor injection, tumor tissues were isolated, flash-frozen in liquid nitrogen or treated in paraformaldehyde for histology. 

### 4.13. Statistical Analysis

Data are expressed as mean±s.e.m. and were statistically evaluated with oneway ANOVA followed by the Bonferroni post hoc test using GraphPad Prism 5.04 software (GraphPad Software). *p* < 0.05 was considered statistically significant.

## 5. Conclusions

We uncovered the first mechanism of YBX1 upregulation at a post-translational stage, which is elicited by AURKA. Similarly, this study also uncovered that YBX1 actually upregulates AR post-translationally, but not by increasing its transcription as reported previously. Furthermore, while life-threatening chemoresistance, EMT and cancer stem cell phenotypes define CRPC, the mechanism by which AURKA might facilitate these events has not been examined. The discovery of YBX1 as both downstream and upstream of AURKA provides a unique mechanism by which these two proteins synergize to facilitate EMT, chemoresistance and CSC phenotypes. Thus, inhibiting both AURKA and YBX1 in combination should be highly effective in reversing chemoresistance, tumorigenesis and metastasis in CRPC. 

## Supplementary Materials

The following are available online at https://www.mdpi.com/2072-6694/12/3/660/s1, Figure S1: Raw data for Figure 2A,C,E,G,M,O of the main manuscript. Figure S2: Raw data for Figure 3A,C,E,G,M,O of the main manuscript. Figure S3: Raw data shows subcellular localization of AURKA and YBX1 in C4-2 and 22Rv1 cells. Figure S3: Raw data for Figure 4E of the main manuscript. Figure S4: Raw data for Figure 6F,H of the main manuscript. Figure S5: Raw data for Figure 7E of the main manuscript. Table S1: The details of the antibodies used in this study. Table S2: The sequences of qPCR primers used in this study. The manuscript includes two supplementary tables and five supplementary files.

## Abbreviations

PCa	Prostate cancer
CRPC	Castration resistance prostate cancer
AR	androgen receptor
EMT	epithelial to mesenchymal transition
CSC	cancer stem cells
