# Supplementary material for: Aurora Kinase A-YBX1 Synergy Fuels Aggressive Oncogenic Phenotypes and Chemoresistance in Castration-Resistant Prostate Cancer
Source: Cancers (Basel). 2020 Mar 12;12(3):660. doi: 10.3390/cancers12030660 (PMC7140108; doi:10.3390/cancers12030660)
Supplement: Supplementary file 1 [file cancers-12-00660-s001.zip › cancers-682123-supplementary-final.pdf]

cancers

MDPI

Article

Aurora Kinase A-YBX1 Synergy Fuels Aggressive Oncogenic Phenotypes and Chemoresistance in Castration-Resistant Prostate Cancer

Kumar Nikhil, Asif Raza, Hanan S. Haymour, Benjamin V. Flueckiger, Jiachong Chu and Kavita Shah

Supplementary Materials

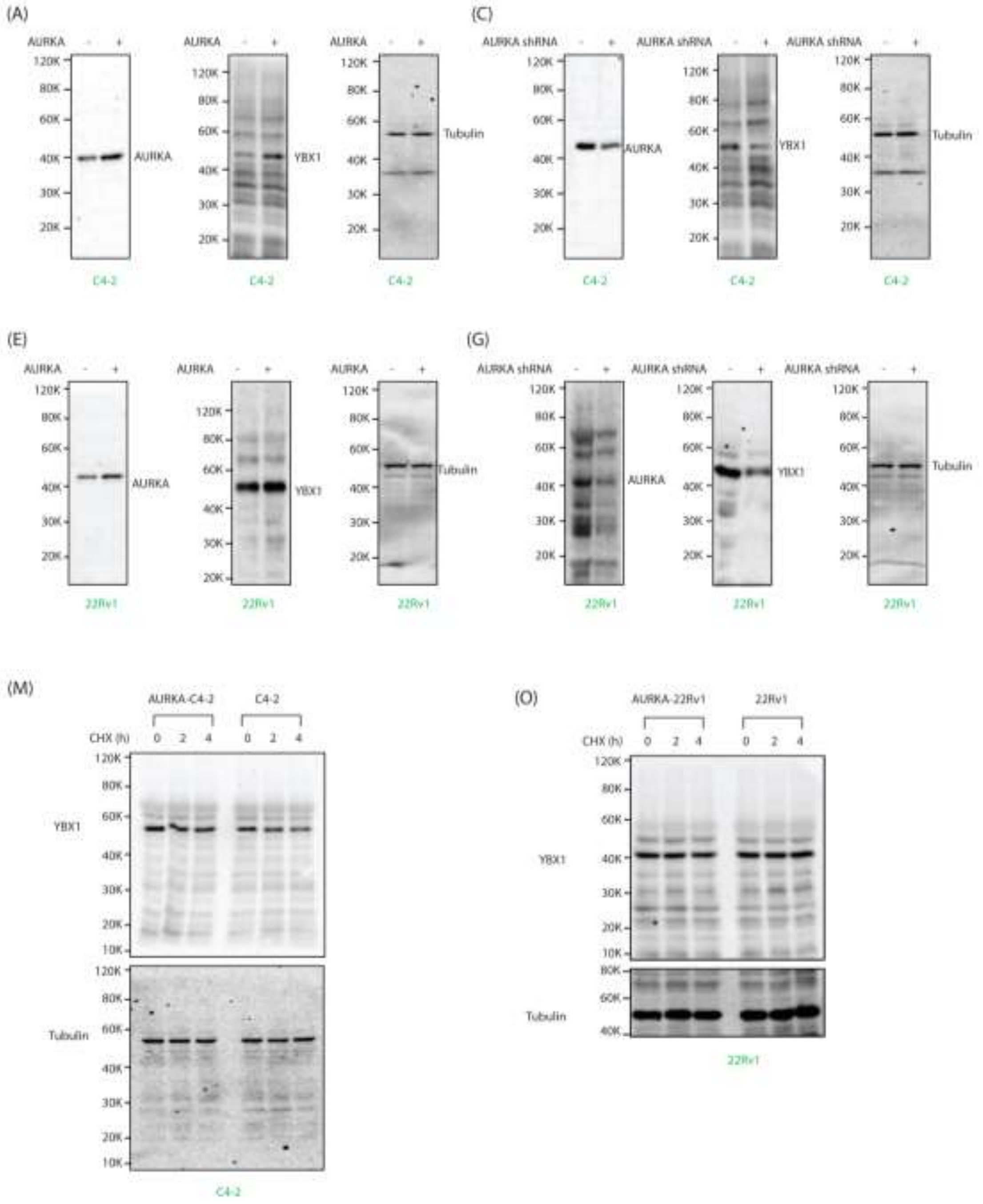

Figure 1. Raw data for figure 2A, 2C, 2E, 2G, 2M and 2O of the main manuscript.

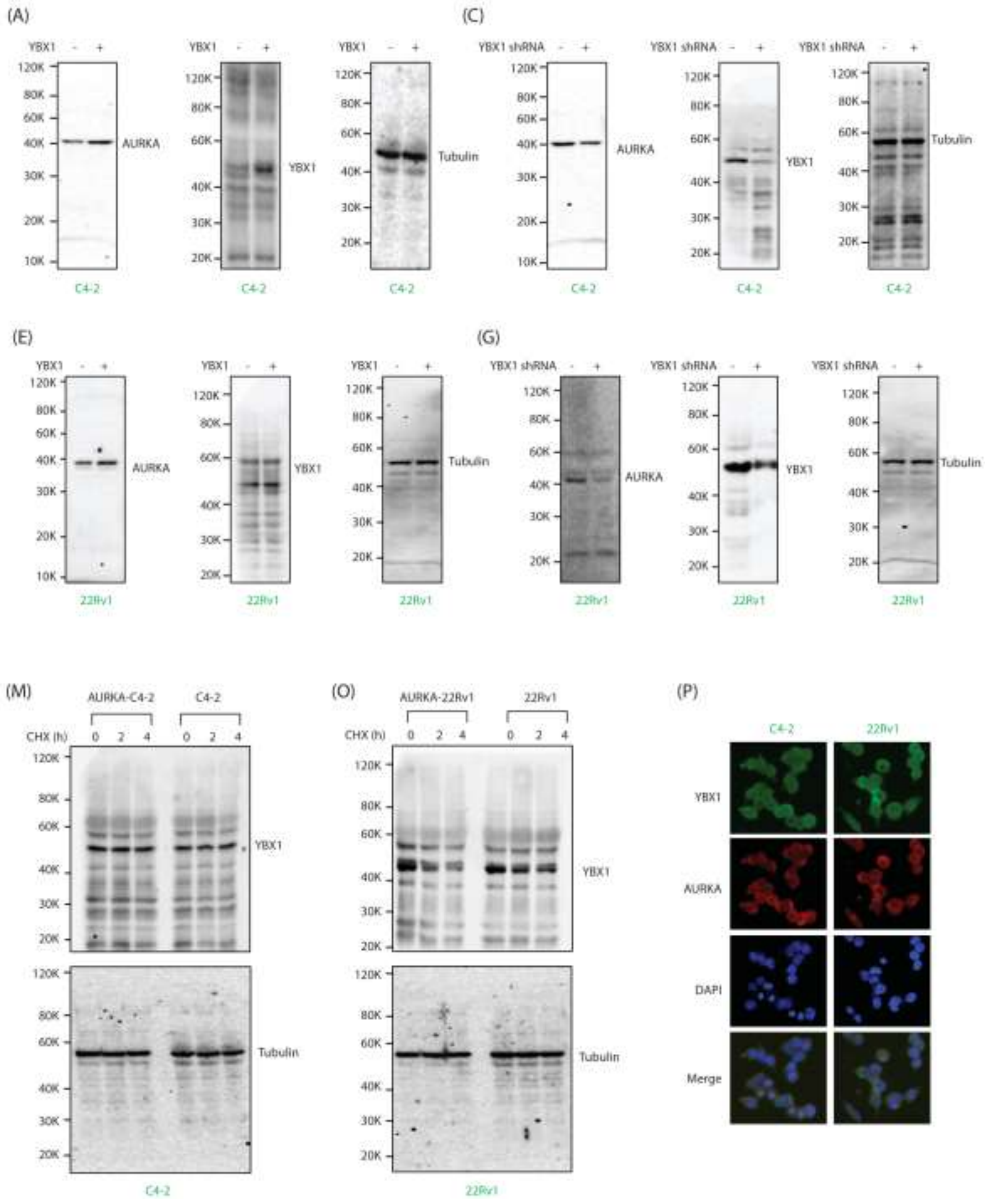

Figure 2. Raw data for figure 3A, 3C, 3E, 3G, 3M and 3O of the main manuscript. Fig. S2P shows subcellular localization of AURKA and YBX1 in C4-2 and 22Rv1 cells.

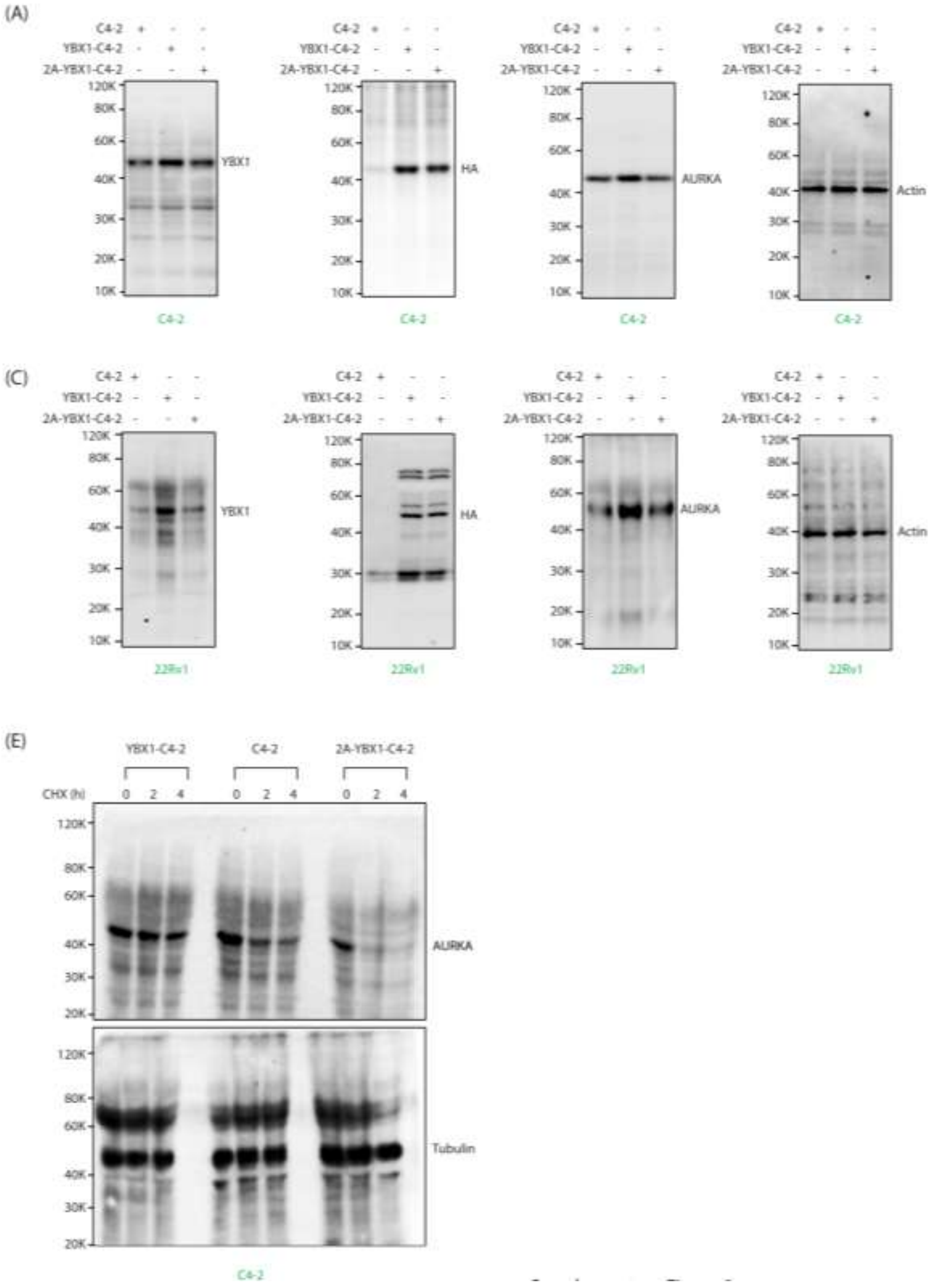

Figure S3. Raw data for figure 4A, 4C and 4E of the main manuscript.

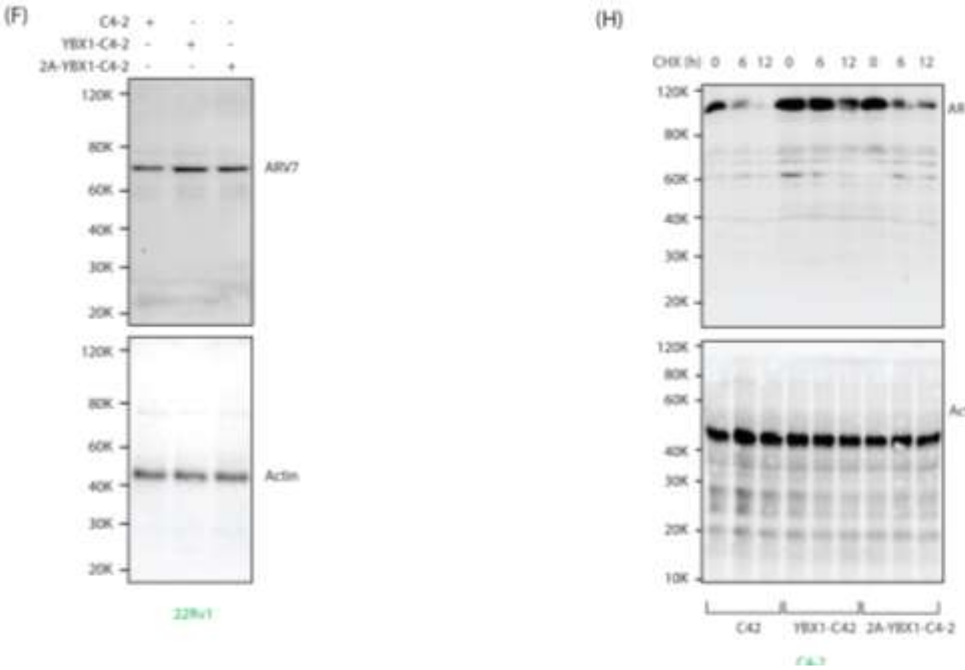

Figure 4. Raw data for figure 6F and 6H of the main manuscript.

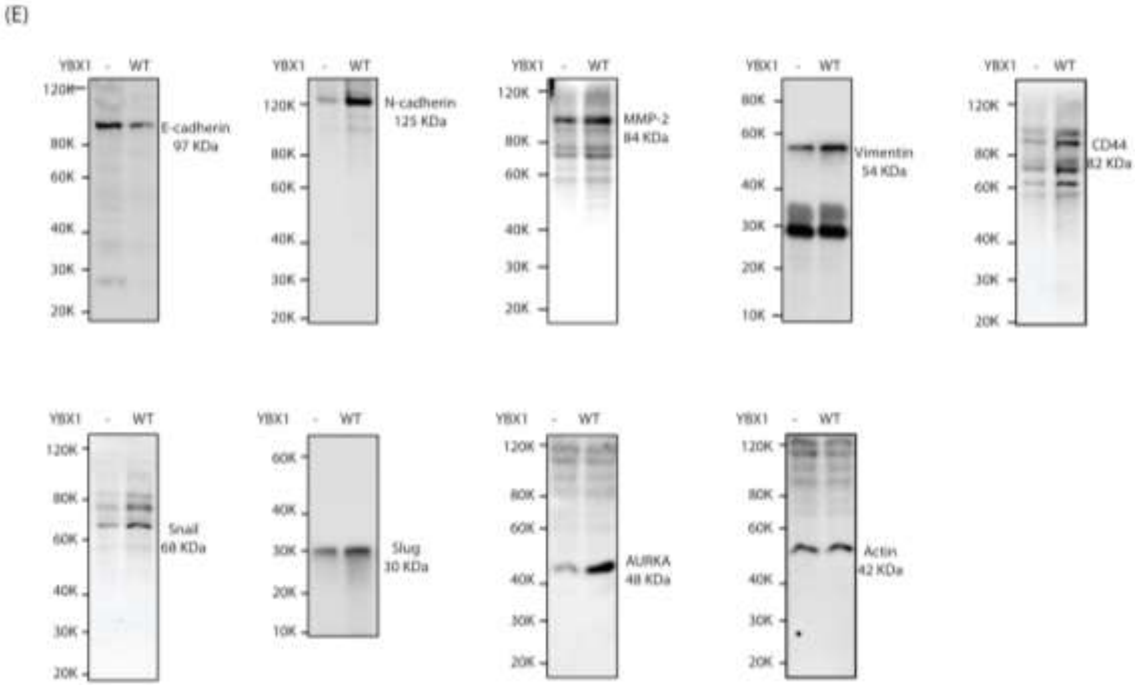

Figure 5. Raw data for figure 7E of the main manuscript.
